# Twin machines validation for VMAT treatments using electronic portal-imaging device: a multicenter study

**DOI:** 10.1186/s13014-015-0577-3

**Published:** 2016-01-14

**Authors:** P. Fenoglietto, M. Khodri, D. Nguyen, F. Josserand-Pietri, N. Aillères

**Affiliations:** Département d’Oncologie Radiothérapie, Institut régional du Cancer de Montpellier (ICM), 208 rue des Apothicaires, F-34298 Montpellier, Cedex 5 France; Département d’Oncologie Radiothérapie, Institut de Cancérologie de la Loire (ICL), Saint Etienne, France; Service de radiothérapie, Groupe ORLAM, Mâcon, France; Service de radiothérapie, Groupe ORLAM, Villeurbanne, France

## Abstract

**Purpose:**

To verify the accuracy of volumetric arc therapy (VMAT) using the RapidArc™ device when switching patients from one single linear accelerator (linac) to a paired energy and mechanics "twin" linac without reoptimization of the original treatment plan.

**Patients and Methods:**

Four centers using 8 linacs were involved in this study. Seventy-four patients previously treated with the 6MV photon RapidArc™ technique were selected for analysis, using 242 measurements. In each institution, all patients were planned on linac A, and their plans were verified both on linac A and on the twin linac B. Verifications were done using the amorphous silicium electronic portal imager (EPID) of the linacs and were analyzed with the EpiQa software (Epidos, Bratislavia, Slovakia). The gamma index formalism was used for validation with a double threshold of 3 % and 3 mm with a measurement resolution of 0.39 mm/pixel, and a smoothed resolution of approximately 2.5 mm.

**Results:**

The number of points passing the gamma criteria between the measured and computed doses was 94.79 ± 2.57 % for linac A and 94.61 ± 2.46 % for linac B. Concerning the smoothed measurement analysis, 98.67 ± 1.26 % and 98.59 ± 1.20 % points passing the threshold were obtained for linacs A and B, respectively.

The difference between the 2 dose matrices acquired on the EPID was very small, with 99.92 ± 0.06 % of the points passing the criteria.

**Conclusion:**

For linacs sharing the same mechanical and energy parameters, this study tends to indicate that patients may be safely switched from treatment with one linac to treatment with its twin linac using the same VMAT plan.

## Background

New radiotherapy techniques aiming at improving target coverage and protection of organs-at-risk have emerged in the last decade. Intensity-modulated radiation therapy (IMRT) improved dosimetric results compared with 3-dimensional conformal radiotherapy (3D-CRT) by modulating the beam intensity for static gantry angles [1–4]. After a development phase followed by a validation process [5, 6], IMRT is now routinely implemented and available for a growing number of patients. New quality assurance procedures had to be designed for IMRT as the complexity of optimization algorithm and technical delivery were increasing. Software tools were developed for quality assurance [7–9] and for treatment planning systems [10, 11]. Guidelines on the technical and clinical aspects of the validation of IMRT treatments were also established [4, 12–15].

Volumetric arc therapy (VMAT), commercialized under the names RapidArc (Varian) or VMAT (Elekta), is a refinement of standard IMRT which uses beam modulation combined with a continuous or discrete arc motion of the linac gantry around the patient. The resulting treatment plan is often more complex than that of the standard IMRT, which raises the question of the feasibility of switching a patient treated with a VMAT plan computed for a specific linac to a “twin” linac e.g. in case of repair or maintenance. While it has been shown possible for 3D-CRT and standard IMRT, no data is available regarding this possibility with VMAT. We thus conducted a study in order to compare the accuracy of the actual patients’ VMAT plans optimized for a specific linac to the same plans when radiotherapy is delivered on a “twin” linac.

## Patients and methods

IMRT was implemented in four centers between 2001 and 2008, and more than 10 000 patients were treated using this technique since. Data was collected on the eight linacs (21 EX and 21 iX, Varian Medical System, Palo Alto, CA) used to deliver IMRT treatments in these centers. They were all equipped with 120 leaves multi-leaf collimators (MLC) adjusted and verified by the medical physicists so as to be equivalent regarding the “sliding-window” delivery technique. Between 2008 and 2010, RapidArc options were installed on the linacs and patients started to be treated with this modality. Otto et al. [16] showed that the RapidArc combines dose-rate modulation, gantry-speed modification and leaf position to create a high-dose modulation during a single or few rotations around the patient. Commissioning of the 8 linacs was carried out following the manufacturer’s suggestions [17]. Given the higher number of parameters to verify for the RapidArc technology compared to the static gantry technique, it was important to know if the patients could be switched from a linac for which the treatment plan had been specifically optimized for a “twin” linac. All linacs were equipped with amorphous silicon aS1000 (Varian) electronic portal imaging devices (EPIDs), which allow portal-imaging dosimetry for IMRT verification [18]. In this study, we used EPIDs to validate the linac matching and to investigate the possibility of switching a patient from one linac to another without the need of re-optimizing a new VMAT plan. At the time of the study, the V8 version of ARIA did not allow the RapidArc portal-dose predictions for comparisons with the image measured on the AS1000. We then decided to use the EpiQa software (Epidos, Bratislavia, Slovakia) for data analysis, based on previous publications [19, 20].

### Patients’ selection and study design

Seventy-four patients previously treated with RapidArc for prostate (*n* = 10), head and neck (*n* = 30), pelvis (*n* = 20) or miscellaneous (*n* = 14) cancers in four French oncology centers (Institut régional du Cancer de Montpellier - ICM, Montpellier, Institut de Cancérologie de la Loire, Saint Etienne, and the ORLAM radiotherapy centers of Mâcon and Villeurbanne) were included in the study between 2008 and 2010, and accounted for 242 measurements. Consecutive patients were selected in each center, independently of the quality assurance results of their pre-treatment plan. Three centers each provided data for 10 prostate or pelvis cases plus for 10 head and neck cancer patients, whereas the fourth center provided the 14 miscellaneous cancer cases. The dosimetric plans were computed using Eclipse treatment planning system for a specific linac (linac A). All the patients’ plans were then verified through a quality assurance process on linacs A and B, the latter having been adjusted by the physics team to be similar during initial commissioning (see “Machine matching”). Rotational treatment plans optimized for linac A were delivered using linac A and linac B on a Silicium detector attached to the gantry. The EPID measured the collapsed plan dose in a 2-dimensional matrix, which was then compared to the one computed in Eclipse on a computer model of the phantom [19, 20]. The gamma index formalism [21] was used for validation with a threshold of 3 % and/or 3 mm, and the number of points reaching these criteria was recorded for comparison. The region of interest corresponded to the entire field with a 1 cm margin. A global gamma comparison method was chosen. Since the anisotropic analytical algorithm calculation grid was 2.5 mm and the measurement resolution was 0.39 mm/pixel, we adopted a smoothing analysis. A Gaussian curve was applied to the measurements in order to decrease the spatial resolution of the matrix to the one computed in Eclipse, resulting in an acquisition matrix of approximately 2.5 mm/pixel. A large range of photon energies (6, 8, 18 and 25 MV) and maximum dose rates (400 to 600 MU/min) were used to analyze if there was specific energy or dose rate dependence of the measurements. A comparison between the dose prediction and dose calculation for linacs A and B was carried out. The results of the plans irradiated with linacs A and B were then compared.

### Treatment planning

Optimization and calculations were done at center one (20 patients) using the 8.0.5 version (PRO I) of the Eclipse treatment planning system (Varian, Palo Alto, USA), which only allowed a single arc. Results for the 10 prostate and the 10 head and neck cases were obtained in late 2008 and early 2009. The three other centers (2 to 4) used the 8.2.23 version of the Eclipse system, which includes the second version of the progressive resolution optimizer (PRO II) and allows an optimization for one or two arcs. Prostate treatments were administered with one arc whereas two arcs were used for other pathologies.

RapidArc optimization was performed using the new PRO algorithm that allows iterative changes to the dynamic delivery variables (i.e. MLC, dose rate and gantry angular velocity) via a set of penalty functions. These iterations were separated into five resolution levels. The first represented the full arc with 10 control points (mostly static fields). This number of control points was then doubled plus one for each successive resolution level, with the final arc including up to 177 control points. As each new control point was added, the dynamic variables were interpolated from the two neighboring points. The nature of this process meant that the lower resolution levels were flexible to optimization objective changes but gave a coarse representation of the full arc, while the higher levels were less flexible but gave a much more accurate representation of the full dynamic arc.

Due to the increased time needed for optimization using PRO I and PRO II, we adopted a strategy where the set of constraints for a specific patient was searched with the dose volume optimizer (DVO). The algorithm, also used for sliding-window IMRT, is based on fluence determination and any change in the constraints has a direct impact on the dose-volume histograms during the optimization. After a few iterations with the DVO, a personalized set of constraints for the specific patients was obtained, which was used to facilitate RapidArc optimization.

As opposed to the DVO algorithm, machine parameters were directly used during optimization with the PRO algorithm. This could have an impact on the final results. Indeed, the DVO algorithm generates a theoretical fluence map at the end of its optimization process, which is converted in an actual fluence by a sequencer including both mechanical and dosimetric linac parameters. The actual dose distribution was then computed on patient data using the predicted fluence and this often resulted in differences between the dose-volume histograms shown at the end of the optimization process and those obtained after the sequencer generated the fluence. With the PRO algorithm, the dose-volume histogram results at the end of the optimization process are usually very close to those obtained after dose calculation. Dose calculation on Eclipse used the anisotropic analytical algorithm with a calculation grid of 2.5 mm.

### Machine matching

Many parameters were checked to consider that two linacs are matched together or form a “twin”-pair. The first concerns relative dosimetric data. Depth dose curves were identical (we applied a threshold of 0.5 %) and special attention was given to small fields, especially when the linacs were used for IMRT treatments. The dose profiles were also checked to be as similar as possible, and a focus on larger fields was necessary since tuning the beam to obtain identical profiles was more difficult for large fields. Dosimetric parameters were primordial factors for the matching procedure. Machine calibration was done in the same conditions and output factors did not differ by more than 0.5 %. After these goals were achieved and beam matching was obtained for 3D-CRT, matching for IMRT also needed a fine MLC tuning and leaf calibration to obtain equivalent results for both linacs. Sharing the same MLC leakage transmission, the calibration of the dosimetric leave gaps was mechanically adjusted to obtain less than 0.5 % difference for the same plan delivered on two different linacs. This method has previously been described in the literature [4, 22, 23]. Furthermore, leaf transmission and dosimetric leave gaps parameters were adjusted in the treatment planning system to obtain similar measurements for the same treatment plan, as described by Chauvet et al. [24].

### Machine tests and patient quality assurance

Delivery measurements were acquired with the EPID of the linacs using amorphous silicium (AS1000) and analysed with the EpiQa software (Epidos, Bratislavia, Slovakia). This program allows the conversion of a dosimetric image acquired by an EPID into a dose map and to compare the dose map with a reference dose distribution. It is possible to use Epiqa for the verification of static and intensity modulated fields.

The portal dosimetry image conversion to dose map was based on the GLAaS algorithm as described by Nicolini et al. [19].

**Fig. 1:**
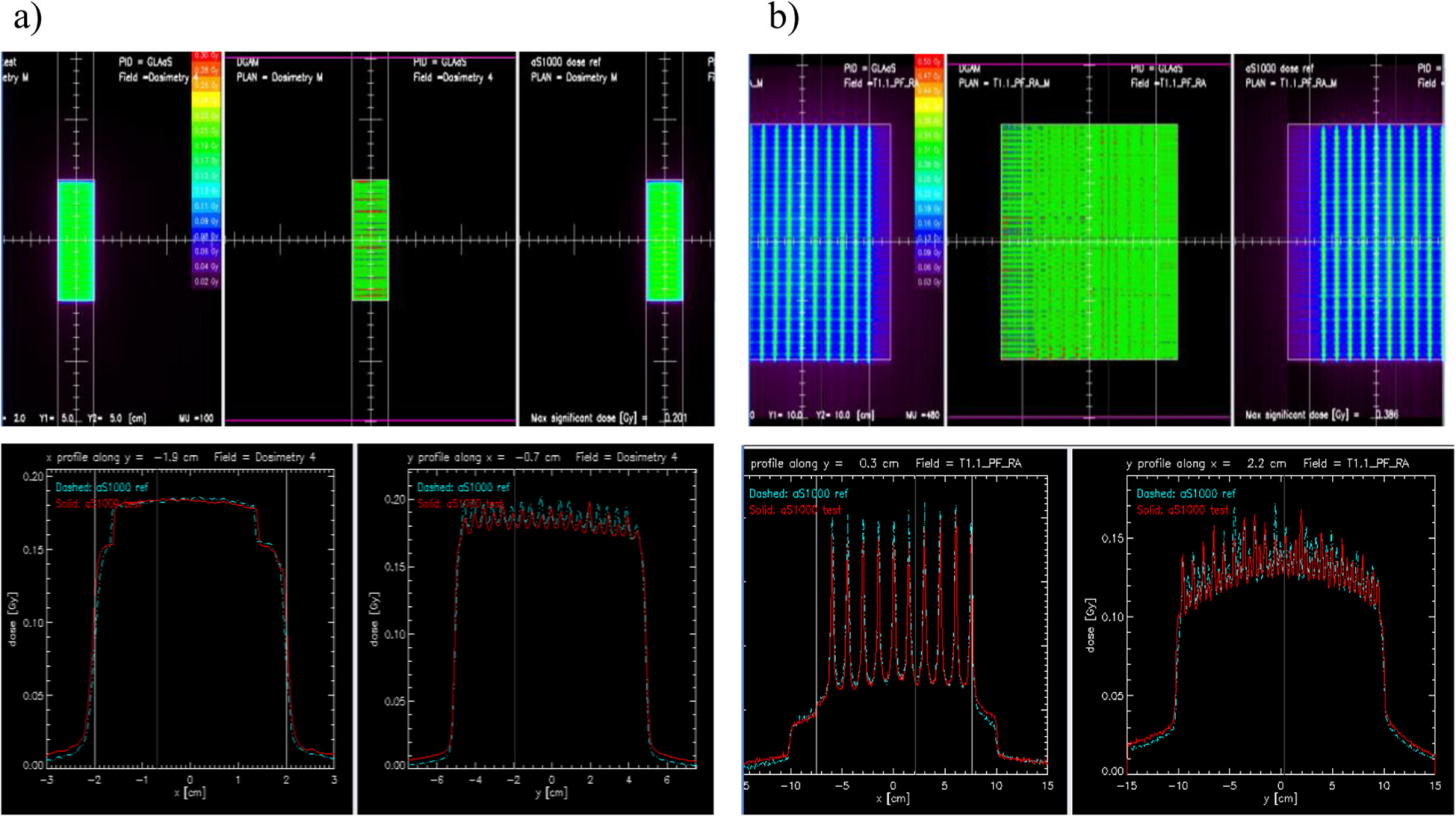
Comparison tests for MLC and EPID calibrations. **a** Comparison of sliding window tests for verification of the dosimetric leave separation; (**b**) picket-fence tests for leave positioning accuracy. The profiles were taken perpendicularly following the cross-hair axes display

Technical quality assurance for linacs A and B was accomplished using the DICOM files provided by Varian and following the process defined by Ling et al. [17]. The accuracy of the leave movements was tested using the “sliding-window” and “picket fence” tests on different gantry positions [22, 23, 25]. The results obtained with linacs A and B were used to evaluate the quality of the matching (Fig. 1). These tests provided a more qualitative than quantitative analysis, and were similar to the film dosimetry quality assurance, which has been used for many years. A garden fence methodology was followed to check the quality of these results [22]. Specific tests were done using the RapidArc to verify the dose-rate, the gantry speed and the leaf speed variations during rotation as to be assured of the consistency of the two linacs.

Regarding the treatment plans, once the dosimetry was finished, a quality control plan was realized using the GlAas algorithm formalism [19], commercially available in the Epiqa software (Epidos, Bratislava, Slovakia). A dose distribution on a virtual water phantom at the depth of the maximum of the depth dose curve and a distance of 100 cm to the source was calculated in Eclipse. This verification plan, using the original parameters (MLC and dose rate) was “collapsed” on an infinitesimal gantry rotation, generating a dose distribution of the whole arc on a single plane, perpendicular to the beam central axis. This dose distribution, calculated with a resolution of 2.5 mm/pixel, was exported and compared with the data acquired on the EPID (AS1000, Varian Medical System, Palo Alto, CA, USA) during the delivery of the real patients’ plan using the Epiqa software. The GlAas algorithm was configured to convert the images acquired without any build-up on the EPID cassette into a dose at the depth of the maximum of the depth dose curve (e.g. 1.4 cm for X6). The measurement consisted of an acquisition of the real treatment plan during the RapidArc delivery using the integrated mode of the EPID. The detector was positioned at the isocenter distance without any patient, phantom or couch inside the beam. [20] Spatial resolution was an important factor for the evaluation of the results. As the detector provided a resolution of 0.39 mm/pixel, lower than the 2.5 mm/pixel grid calculation in Eclipse, we performed a second analysis using a Gaussian smoothing method of 2 mm provided by the Epiqa software, in order to have a comparison with similar spatial resolution. Our tolerance level for acceptance of a treatment plan was a 3 %-3 mm gamma index threshold with an acceptance value of 95 % of the point passing these criteria. For cases below the 95 % acceptance level, qualitative analysis could still lead to a quality assurance validation. Using this data, we first compared linacs A and B of each center calculating the difference between dose-calculation and dose-measurement for the same treatment plan. Second, we compared the treatment delivery using the measurements made with linac A to those made with linac B. This evaluation excluded all treatment planning system algorithm configurations and was purely an indicator of the linac beam, MLC and EPID parameters calibrations.

## Results

Analysis of the “sliding window” and the “picket fence” tests were made qualitatively by looking at the curve correspondence on portal measurements. It showed a good agreement between linacs A and B (Fig. 1). We took a series of mechanical and imaging tests of the MLC using the same DICOM file on the twin linacs, showing equivalent results. First, the sliding window IMRT files were used to measure absolute dose with an ionisation chamber in a PMMA phantom to ensure proper calibration of the leaves on each MLC. Results were within 1%. We then performed portal IMRT tests (e.g. chair, step wedges or pyramid) and the difference for a gamma 3 %-3 mm between the 2 linacs was less than 1 %. The specific RapidArc quality assurance test results were concordant with those described by Jorgensen et al. [26].

**Fig. 2:**
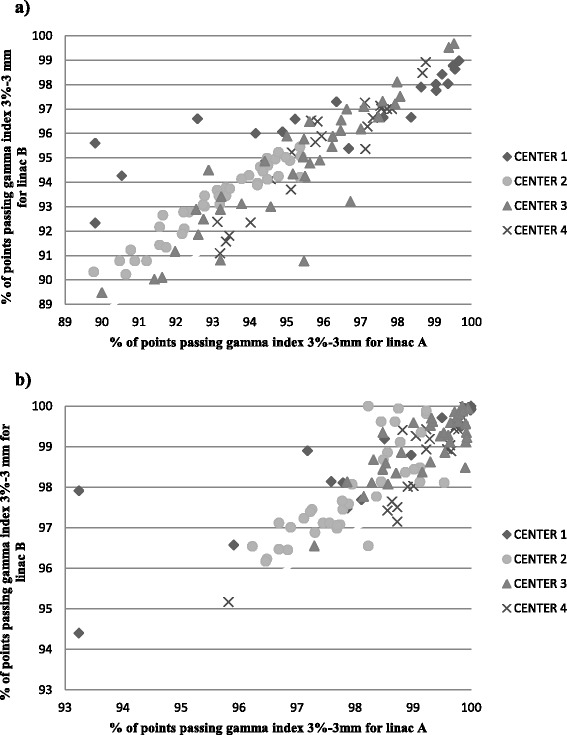
Global comparison of the predicted and effective dose distribution for the twin linacs with (**a**) and without (**b**) smoothing

Concerning the patients treatment plans, the comparison between measured and computed dose showed 94.79 ± 2.57 % of points passing the gamma criteria for linac A and 94.61 ± 2.46 % for linac B (Fig. 2a). For the smooth analysis, we reached 98.67 ± 1.26 % and 98.59 ± 1.20 % for linacs A and B, respectively (Fig. 2b). Regarding the ten head and neck patients, the results were 94.03 ± 2.08 % and 94.20 ± 2.10 % with a measurement grid of 0.39 mm for linacs A and B, respectively. Once again, a smoothed analysis improved the results: 98.51 ± 1.39 % (A) and 98.59 ± 1.12 % (B) of points passing a gamma ≤1 for 3 % or 3 mm.

**Fig. 3:**
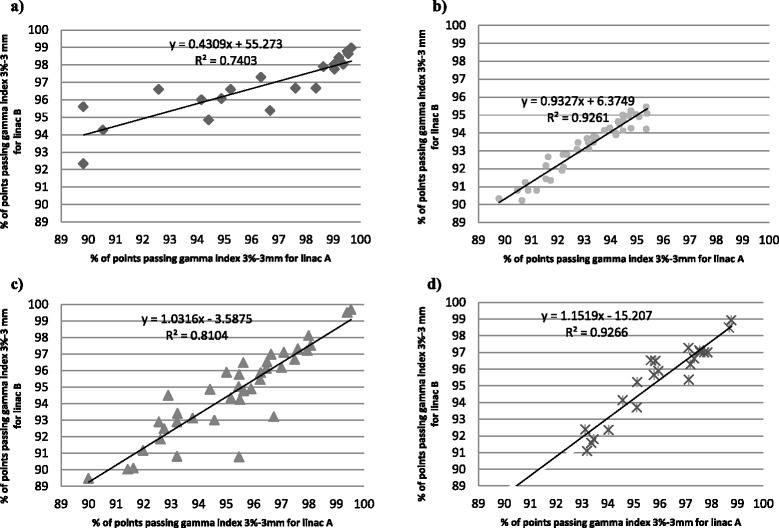
Comparison of the predicted and effective dose distribution for the twin linacs by center. The X- and Y-axes represent the gamma index result for a 3 %-3 mm analysis for linacs A and B, respectively, for **a**) center 1, **b**) center 2, **c**) center 3 and **d**) center 4

**Fig. 4:**
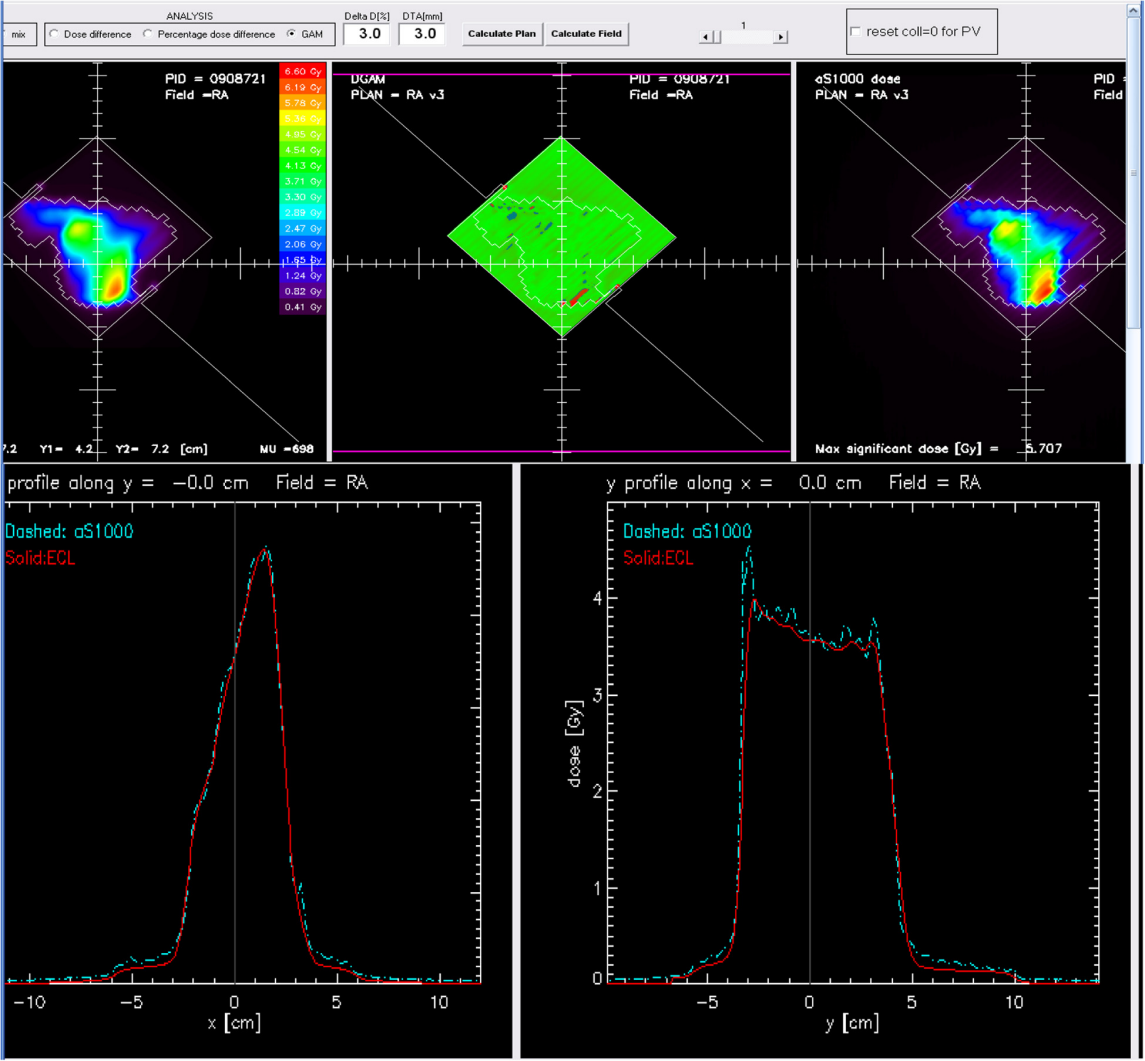
Over-modulation on measurement before smoothing. Profiles were taken perpendicularly following the cross-hair axes display

For the prostate cancer patients, for whom the modulation was much simpler, the results were 99.00 ± 0.48 % for linac A, and 97.98 ± 0.59 % for linac B without smoothing even for the plans optimized using the PRO version (center 1). The results using the smoothing method were 99.77 ± 0.31 % and 99.84 ± 0.16 % for the linacs A and B, respectively. For patients with pelvic cancer, results were 94.13 ± 2.44 % and 98.26 ± 1.19 % for linac A, and 93.99 ± 231 % and 98.12 ± 1.23 % for linac B, before and after smoothing, respectively. For centers 2, 3 and 4, the quality assurance results on linac A compared to those on linac B were good with a maximum difference of 1.9 % in the values obtained (Fig. 3b, c, and d). For center 1, the maximal difference was 6.4 % for a head and neck treatment delivered with a single arc. This difference can be explained by the specificity of verification using collapsed plans on 2D views and because of an over modulation measured by the portal imager (peaks on the blue line on Fig. 4), especially for this complex case with a high modulation level. Adding a second arc decreased the modulation level by arc, and consequently allowed an improved result.

**Fig. 5:**
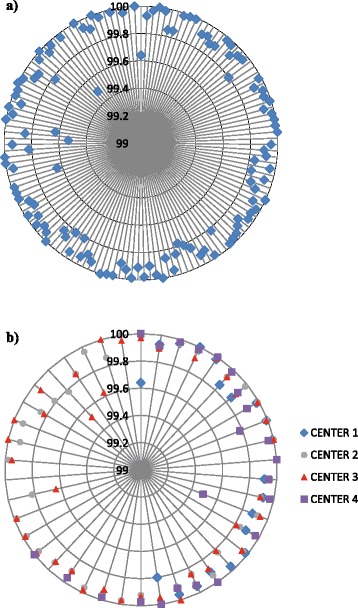
Comparison of the dose measurements between the twin machines: global (**a**) and per center (**b**) results. Each dot on a ray represents a value for patient measurement. The percentages of points passing a gamma index of 3 %-3 mm are represented by the different circle levels

All plans but one passed the criteria of 3 % - 3 mm for 95 % of the points analyzed with the smooth evaluation for the twin machines in the four institutes (Fig. 2b).

Regarding the dose distribution comparison test for the same treatment plans acquired on different portal devices, the differences between linacs A and B were very small, 99.92 ± 0.06 % of points passing the criteria (Fig. 5a). In this analysis, raw data with a 0.39 mm/pixel resolution was used. This test is the most robust to compare two matched linacs and showed excellent results for all the participating centers (Fig. 5b).

## Discussion

IMRT has the ability to deliver highly conformal dose distributions to complex targets. In both static and rotational IMRT, many parameters such as the leaf position, dose rate and gantry speed have to be accurately controlled by the treatment planning system in order to achieve such coverage. Consequently, IMRT quality assurance procedures have to be specifically developed, using ionisation chambers, films or a 2D array [12, 27]. Although VMAT represents an evolution of IMRT, the quality assurance process remains similar. Films and 2D arrays are mostly used to compare the predicted and measured doses, with results reaching the acceptation criteria usually admitted [28–31]. More recently, the use of EPIDs has made the quality assurance easier to set up by taking out the need to use phantoms [32]. This methodology, concerning IMRT quality assurance, was adopted by many institutions [20, 32–35] Validation of RapidArc treatments with EPIDs on 3 different matched beam using Varian linacs was previously described by Fredh et al. [36]. The effect of the gantry movement was not found to significantly affect the results leading to the use of EPIDs as a detector even in the rotational mode. The results were quite similar even after segmenting the 360° arc into multiple sub-arcs of 6° and 12° to “collapse” the irradiation into a 2D measurement [20]. The consistency and reproducibility of VMAT plan delivery were shown with 3 different quality assurance systems by Chandraraj et al. [37].

Our study focused on the capacity of twin linacs to deliver the same dose distribution from the same VMAT plan. The algorithm version of the optimiser was not included in this comparison; only dosimetric and mechanical tunings of the linacs were compared. Our results showed good and equivalent results for all the centers, allowing the possibility for a patient to be switched from one linac to another without the need of repositioning a new plan, provided that the beams and MLC are perfectly matched.

The percentage of points passing the gamma criteria of 3 %-3 mm were similar to other studies using EPID or films for quality assurance [38, 39], even if another study has reported better results [40]. The value of the gamma index highly depends on the spatial resolution used for the analysis. Most of the publications showing overall gamma values better than 99 % have used 2D matrixes with a spatial resolution ranging from 5 to 10 mm, whereas EPIDs have a sub-millimeter resolution of 0.39 mm/pixel. We performed a smoothed analysis to render the resolution closer to a calculated planar dose. Doing so, the results of the gamma index were significantly improved. Another method could have been to increase the spatial resolution of the calculation grid in Eclipse to the lowest value proposed of 1 mm, but the commercial hardware actually available could not carry out this calculation on a real patient volume.

## Conclusion

This study carried out on 8 linacs in 4 centers and for different cancer types tends to indicate that it is possible to treat a patient with the same VMAT plan on twin linacs, provided that the linacs are perfectly matched.
